# Dispersal Timing: Emigration of Insects Living in Patchy Environments

**DOI:** 10.1371/journal.pone.0128672

**Published:** 2015-07-01

**Authors:** Milica Lakovic, Hans-Joachim Poethke, Thomas Hovestadt

**Affiliations:** 1 Field Station Fabrikschleichach, Department of Animal Ecology and Tropical Biology, Biozentrum-University of Würzburg, Würzburg, Germany; 2 Department of Biology (TEREC), Ghent University, Ghent, Belgium; Australian National University, AUSTRALIA

## Abstract

Dispersal is a life-history trait affecting dynamics and persistence of populations; it evolves under various known selective pressures. Theoretical studies on dispersal typically assume 'natal dispersal', where individuals emigrate right after birth. But emigration may also occur during a later moment within a reproductive season ('breeding dispersal'). For example, some female butterflies first deposit eggs in their natal patch before migrating to other site(s) to continue egg-laying there. How breeding compared to natal dispersal influences the evolution of dispersal has not been explored. To close this gap we used an individual-based simulation approach to analyze (i) the evolution of timing of breeding dispersal in annual organisms, (ii) its influence on dispersal (compared to natal dispersal). Furthermore, we tested (iii) its performance in direct evolutionary contest with individuals following a natal dispersal strategy. Our results show that evolution should typically result in lower dispersal under breeding dispersal, especially when costs of dispersal are low and population size is small. By distributing offspring evenly across two patches, breeding dispersal allows reducing direct sibling competition in the next generation whereas natal dispersal can only reduce trans-generational kin competition by producing highly dispersive offspring in each generation. The added benefit of breeding dispersal is most prominent in patches with small population sizes. Finally, the evolutionary contests show that a breeding dispersal strategy would universally out-compete natal dispersal.

## Introduction

Dispersal is an important life history trait that can strongly affect population dynamics and has profound eco-evolutionary consequences [1]. This especially holds in changing environment where species are confined to increasingly fragmented landscapes and where movement between local populations can affect the persistence and the dynamics of whole meta-populations [2]. Hence, understanding dispersal has received much attention in experimental as well as theoretical research [3].

The tendency to disperse evolves under the influence of various ultimate causes [4] including (avoidance of) kin competition [5, 6, 7, 8, 9, 10, 11], inbreeding avoidance [12, 13, 14, 15], and the spatial and/or temporal variability that affects attributes like demography [16, 17, 18, 19], habitat quality [18, 20]or habitat persistence [21].

Further, dispersal decisions are typically influenced by external factors (abiotic environment, biotic interactions) and the internal state of the organism, i.e. emigration decisions are, presumably, not just random [22]. Particularly, population density can be an indicator of local competition and thus reduced resource availability. Hence, there are clear fitness benefits of leaving a crowded patch (or otherwise poor habitat) but staying in a low-density patch [23], if an individual possesses corresponding information: Migration from a high-density patch to another one increases (on average) fitness expectations of offspring. There is indeed much empirical research confirming density dependent dispersal in butterflies [24, 25, 26, 27], spiders [28, 29] and other insects [30, 31, 32, 33].

Selective pressures outlined above could not only impact the individuals’ propensity to emigrate as such but also the timing of emigration. The exact timing of dispersal is especially important, because whether an individual moves before, during, or after a reproductive episode impacts population dynamics [34]. Regarding timing, two general types of dispersal have been distinguished in empirical as well as theoretical research, 'natal dispersal' and 'breeding dispersal' (also 'adult dispersal', [35]). Natal dispersal occurs if an individual permanently leaves its natal site before ever reproducing. It has been observed in a broad spectrum of animal groups like spiders [36], insects [37], reptiles [3], birds [38] and mammals [39, 40]. Breeding dispersal, on the other hand, considers movement between successive sites of reproduction. Such repeated dispersal has the obvious consequence of distributing life-time reproduction over two or more sites. Usually such dispersal is assumed to occur between reproductive seasons, like in long-lived organisms such as birds or mammals [38, 41]. Consequently, breeding dispersal is an aspect mostly ignored in the models for the evolution of dispersal in annual organisms where dispersal is typically implemented as natal dispersal [16, 17, 18, 42, 43].

However, an effect comparable to breeding dispersal in perennial organisms would emerge in annual organisms that go through only one reproductive cycle but where individuals migrate within the reproductive phase. For example, a female butterfly can first deposit some eggs in the natal patch and then migrate to other site(s) to continue egg-laying there. How often such 'spatially distributed' allocation of reproductive effort within a season occurs in short-lived, seasonal animals is hard to evaluate. Indeed, it is not a trivial exercise to provide such evidence as it requires accurate assessment of the timing of emigration in the field (it is not sufficient to just prove that an individual moved from one site to another); typical mark-recapture studies, for example, do not provide such evidence.

The benefit of distributing offspring in space—either within or across reproductive seasons—has been explained as 'risk-spreading' [44]. Risk spreading may emerge as a response to environmental uncertainty because distributing offspring over several patches could reduce the variance in reproductive success and thus increase the geometric growth rate [45, 46]. Such behavior may especially be important for the persistence of species living in fragmented habitats where risk-spreading can save isolated populations form extinction and secure the persistence of the whole meta-population [46, 47, 48].

However, at the population level there is no fundamental difference between natal dispersal where e.g. 20% of the individuals disperse after birth and breeding dispersal where all individuals produce 80% of their offspring in the natal patch before emigrating and—provided they survive dispersal—producing another 20% of offspring somewhere else. It is thus an open question to what degree different net-emigration would evolve under the natal versus the breeding dispersal strategy and to what degree one of the strategies is superior (in terms of long-term fitness) to the other.

Therefore, the main goal of this paper is to model and quantitatively compare the evolution of natal and breeding dispersal in annual organisms. More specifically, we will evaluate evolution in four fundamentally different scenarios (all combinations of either density-dependent or independent and of either natal or breeding dispersal) and utilize evolutionary tournaments [43] to identify whether and under which conditions one strategy would be able to outcompete the other.

## The Model

### Population dynamics and life-cycle

Our individual-based simulation experiments are based on the model of insect dispersal in patchy landscapes originally published by Poethke and Hovestadt (2002). Landscapes are implemented as a predefined number of *N* habitat patches, each of them with the same mean carrying capacity (*K*).

We consider discrete non-overlapping generations of asexual organisms. In approximation of the life-cycle of insects like grasshoppers or butterflies, we assume that individuals emerge at the start of the season as adults. Adults may either disperse right after emergence ('natal dispersal') or during the reproductive season ('breeding dispersal'). Each individual is characterized by affiliation with a certain patch *i*(initially the natal patch), the dispersal strategy it follows (natal *S*_*N*_ or breeding dispersal *S*_*B*_), and a parameter related to its dispersal strategy; more details on the dispersal process will be provided later.

For any individual, the number of offspring produced is drawn from a Poisson distribution with mean **λ**; as will be explained in more detail below these offspring may, under breeding dispersal, be produced in different habitat patches. Adults die after completion of the reproductive phase. In agreement with the model of Poethke and Hovestadt (2002) survival to adulthood (*s*_*i*,*t*_) in the next generation (*t*+1) of the *L*_*i*,*t*_(*L*_*i*,*t*_ = N_i,t_×λ) larvae produced in patch *i* and generation *t* is density dependent according to the Beverton-Holt model [49]:

$$s_{i,t}=\frac{1}{1+\frac{a\cdot L_{i,t}}{\lambda }}\text{ with }a=\;\frac{\lambda -1}{K_{i,t}}$$ (1)

*K*_*i*,*t*_ is the carrying capacity of a patch *i*in generation *t*. To account for random influences like inter-annual fluctuations in patch quality, *K*_*i*,*t*_ is a log-normal distributed random number with mean *K* and standard deviation *σ*_*K*_.

In accordance with the model of Poethke and Hovestadt (2002) we also tested simulations with inter-annual fluctuations in fertility (*λ*), as might result from e.g. varying weather conditions during egg-laying. This alternative implementation of environmental stochasticity had no qualitative influence on our results.

### Dispersal

The original model by Poethke and Hovestadt (2002) only allowed for natal dispersal (*S*_*N*_) where an individual decides right after birth to emigrate or not. To implement breeding dispersal we modified this model in a sense that individuals were allowed to disperse after spending a fraction *t*_*N*_ of their reproductive life in the natal patch, and then spend the remaining fraction *t*_*E*_ = 1-*t*_*N*_ in another patch. Consequently an individual produces a fraction λ × t_N_ of their offspring in the natal patch and λ × t_E_ in another patch. For example anindividual spends 80 percent of life in its natal patch, thus leaves 80%of its offspring in the natal patch (λ × 0.8)and consequently leaves remaining 20% of offspring in a new patch, given that it survives dispersal (λ × 0.2). However, in both scenarios emigrants face a certain risk of mortality (*μ*) in which case individuals are immediately removed from the population (without reproducing in the target patch). For simplicity we allow for only one dispersal event during an individual's lifetime, i.e. it can maximally distribute its offspring over two different patches.

**Fig 1:**
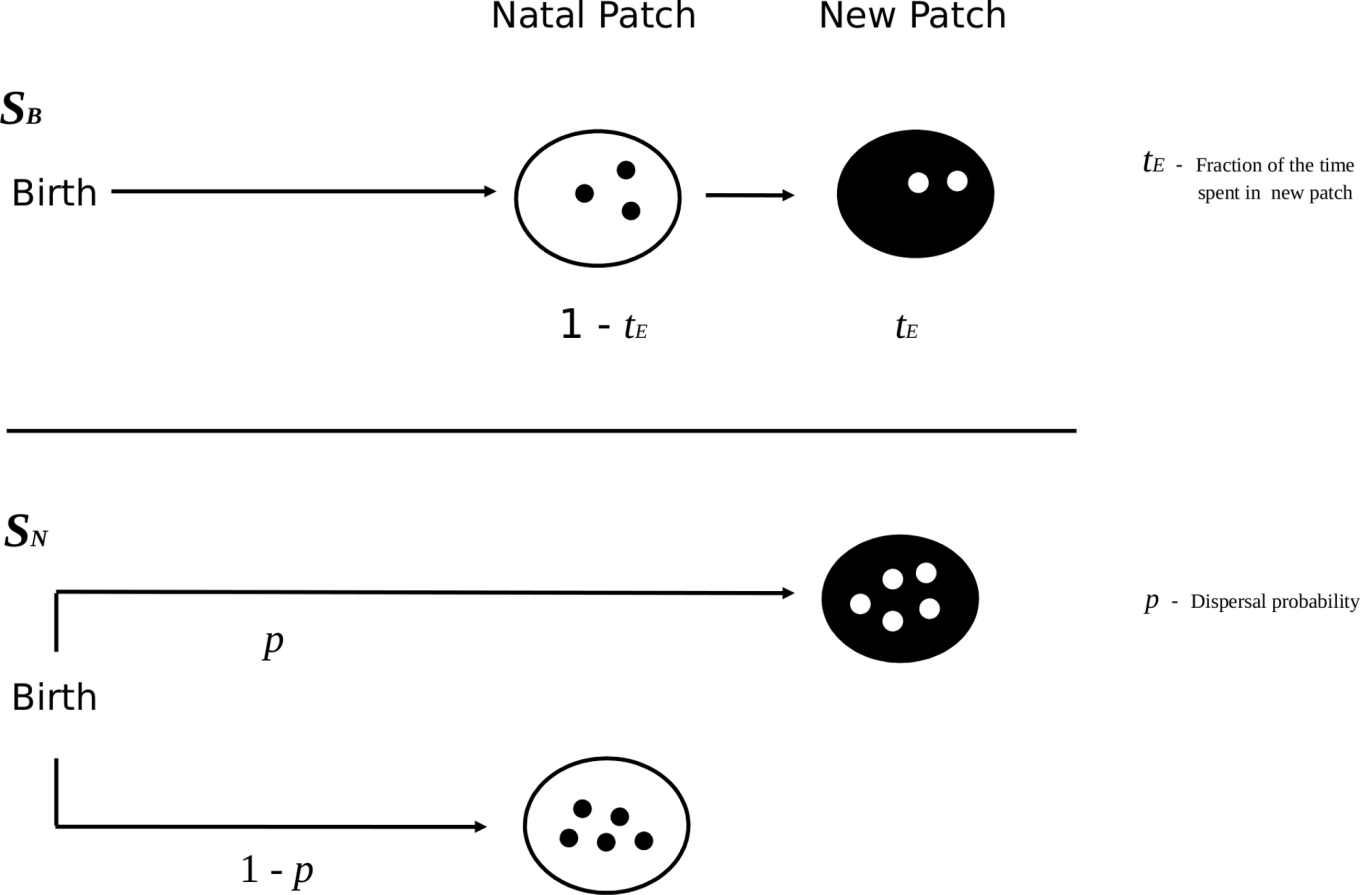
Schematic representation of the breeding *S*_*B*_ and natal dispersal strategy *S*_*N*_. Under *S*_*B*_ reproduction takes place before and after dispersal with a fraction 1-*t*_*E*_ of offspring allocated to the natal patch and a fraction *t*_*E*_ to the target patch. In contrast, under *S*_*N*_ all reproduction always takes place either in the natal patch (with probability 1-*p*) or in the target patch (with probability *p*). In both scenarios dispersing individuals carry a certain mortality risk *μ* during dispersal—in case of mortality individuals will not reproduce in the target patch.

In case of density independent emigration both dispersal strategies can be characterized by (i) an individual's dispersal propensity *p* (emigration probability) and (ii) the fraction of time (*t*_*E*_) spent in another patch after dispersal: Natal dispersal (*S*_*N*_) is implemented by fixing *t*_*E*_ = 1 but allowing emigration probability d = *p* to evolve in the range 0 ⩽ *p* ⩽ 1, whereas breeding dispersal (*S*_*B*_) is implemented by fixing *p* = 1 but allowing d = *t*_*E*_ to evolve in the range 0 ⩽ *t*_*E*_ ⩽ 1(Fig 1).

For both scenarios we also performed simulation experiments with density-dependent emigration, as well: Density dependence was modeled according to Poethke and Hovestadt (2002) with

$$d=\{\begin{array}{cc} 0 & if\;C_{i,t}<C_{T}\\ 1-\frac{C_{T}}{C_{i,t}} & if\;C_{i,t}\geq C_{T} \end{array}\;\text{ with }C_{i,t}=\frac{N_{i,t}}{\bar{\text{K}}}\text{ the population density in patch }i\text{ at time }t\;\;$$ (2)

Here the specific threshold density *C*_*T*_defines either d = *p*(*S*_*N*_) or d = *t*_*E*_(*S*_*B*_)according to Eq (2). Below this threshold density individuals do not disperse.

Offspring inherit their dispersal trait from the parent. However, with a probability of *m*_*R*_ = 0.001 the evolvable trait (either *p* or *t*_*E*_in the case ofdensity independent scenario or *C*_*T*_ in density dependent scenario) may mutate: In this case we add a random value drawn from the uniform distribution [-0.01 to 0.01] to the parent's trait value.

Dispersal is global; dispersing individuals randomly move to one of the *N* patches in the landscape, including its natal patch. As mentioned earlier, we impose a dispersal cost (*μ*) upon all emigrants regardless of the patch origin. This cost can be considered as a probability of mortality during the transitional phase of dispersal, e.g. death by predation or from exhaustion [50].

### Initialization and parameters tested

Simulations were initialized with *K* individuals in each patch. At initialization we assigned different values for the evolvable trait *C*_*T*_ (density dependent dispersal) or *d* (density independent natal orbreeding dispersal) to each individual drawn from a uniform distribution (*C*_*T*_ ∊*∊*[0.6, 1.4]; *d*∊*∊*[0, 1]).

Our model includes three forces selecting for or against dispersal: (i) kin competition, (ii) spatio-temporal heterogeneity, (iii) cost of dispersal. To test for the influence of these forces on the evolution of dispersal we repeated simulations for different parameter settings (*K*, *μ*,*σ*): (1) To keep overall metapopulation size comparable we ran simulations with either a large number (*N* = 1000) of low capacity (*K* = 10) patches or a small number (*N* = 100) of high capacity (*K* = 100) patches. (2) Previous studies have already confirmed that environmental variation selects for higher dispersal [51, 52]. For simplicity and because this is not the major focus of this study we implement here only two extreme scenarios for environmental fluctuations: No fluctuations (*σ* = 0) or very high fluctuations (*σ* = *K*). (3) We further ran simulations covering a broad range ofdispersal mortalities *μ*∊[0.001; 0.01; 0.02; 0.05; 0.1; 0.2; 0.5]. In all scenarios presented here we kept mean fecundity fixed at *λ* = 2; we tested higher values that gave, qualitatively similar results. Mutation rate (*m*_*R*_ = 0.001) and size*m*_*S*_ ∊[-0.01, 0.01] were also kept constant across all simulations.

### Simulations and data extraction

For each the 2x2x7 possible parameter combinations of *K*, *σ*,*μ* and any of the four different dispersal models we performed 15 replicate mono-culture simulation experiments, each running over 7000 generations. Only data from the last 2000 generations—after simulations had reached an evolutionary equilibrium—were utilized for data evaluation. For this period we calculated for every 10th generation the mean dispersal rate across the whole meta-population. The mean dispersal rate at the population level $\overset{-}{d}$ is defined by the mean dispersal propensity ($\overset{-}{d}=\overset{-}{p}$) in the case of natal dispersal (see also Poethke and Hovestadt 2002), while it is equal to the average fraction of time spent away from the natal patch ($\overset{-}{d}=\overset{-}{t_{E}}$) in the case of breeding dispersal. For presentation in figures averages were taken over the last 2000 generations of all 15 replicate simulation runs.

We performed additional non-evolutionary simulationsin order to compare how mode of dispersal affects the formation of the coefficient of relatedness (*F*). For this purpose we fixed the dispersal traits of all individuals to identical values (*d* ∊[0.5, 0.05]) for both strategies, settingdispersal costs (*μ* = 0) in all simulations;After allowing for the population to reach ecological equilibrium (100 generations) we then marked all individuals from a single randomly selected patch with a neutral marker. We then calculated coefficient of relatedness *F* for the individuals carrying this neutral marker after 5 and 20 generations. Rousset [53] defines. *F* in structured populations as:

$$F=\frac{\left(Q_{w}-Q_{b}\right)}{1-\;Q_{B}}$$ (3)

Here *Qw*is the probability of identity within a ‘structural unit’ (patch in our case) and *Qb* the probability of identity between two different patches. To link relatedness *F* to frequency *p* of a neutral marker within the entire metapopulation we can follow the logic well known from the derivation of the Wahlund effect [54]: The mean degree of homozygosity of subpopulations exceeds homozygosity in the entire population by twice between-patche variance in *p*_*i*_ (V[p]). From that it can easily be concluded that:

$$F=\frac{V\left[p\right]}{p\times \left(1-p\right)}$$ (4)

### Evolutionary contest

To compare whether one of the dispersal strategies (*S*_*N*_, *S*_*B*_) would have an evolutionary benefit over the other in direct competition we performed 'evolutionary tournaments' between natal (*S*_*N*_) and breeding dispersal (*S*_*B*_) similar to those described by Hovestadt et al. (2010). For the tournament we initialized 'mixed meta-populations' by introducing 50% of individuals applying strategy *S*_*N*_ according to the distribution of parameter values that had established at the end of the previously introduced monoculture experiments and sampling the remaining 50% from the final parameter distribution as it emerged in the corresponding *S*_*B*_ monoculture experiments. For each contest and parameter combination the tournament was replicated 10 times and we recorded for each tournament whether and after which time one strategy completely outcompeted the other, meaning that one of the strategies went extinct.

## Results

### Natal vs. breeding dispersal

**Fig 2:**
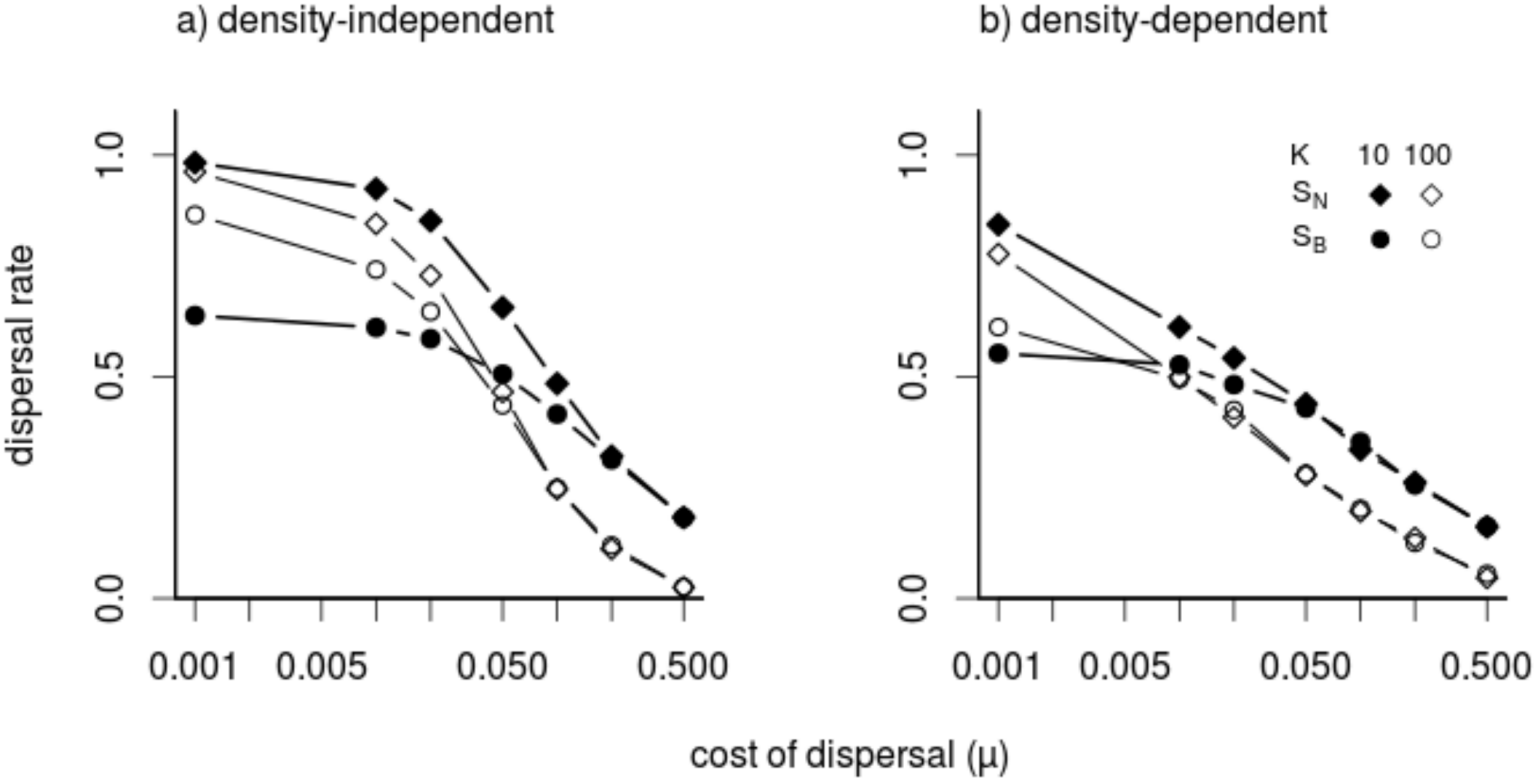
The effect of patch capacity *K* and dispersal strategy on evolved mean dispersal as a function of dispersal mortality,*μ*(log scale). (a) Density-independent (*DI*) scenario and (b) density-dependent (*DD*) scenario. Filled circles and diamonds represent small patches (*K* = 10) and empty circles and diamonds big patches (*K* = 100). Diamonds indicate natal dispersal (*S*_*N*_), circles breeding dispersal (*S*_*B*_). Other parameter values: environmental variability (*σ* = *K*)) and fecundity (*λ* = 2).

In general and expectedly (see discussion), increasing costs of dispersal tends to select against dispersal. Our results confirm previous findings [9, 18], regardless of whether dispersal is density-independent or -dependent and whether it is natal or breeding dispersal (Fig 2). With high mortality risk during dispersal, dispersal is low and more or less equal rates evolve for natal and breeding dispersal. However, as dispersal mortality decreases (c. *μ*< 0.05), an apparent difference between the two strategies arises: Populations following breeding dispersal*S*_*B*_evolve substantially lower dispersal rates than those following natal dispersal (Fig 2). More specifically, even at low dispersal costs, the proportion of offspring dispersed for *S*_*B*_ hardly increase above 0.5–0.6. At such value theoffspring of a successfully dispersing parent are almost equally distributed between the natal and a new patch. It is indeed obvious (if costs of dispersal are low) that kin competition among siblings would be minimized if a parent would distribute its offspring evenly over two patches. However, under natal dispersal, when dispersal mortality is low, emigration probabilities evolve to values close to1. This is because the kin competition reducing benefit of dispersal for the natal dispersal strategy only emerges in the generation of grandchildren (second generation) and beyond, while the breeding dispersal strategy can already reduce direct sibling competition in the first generation. This provides a strong incentive for the evolution of a residence time *t*_*E*_ near 0.5.

**Fig 3:**
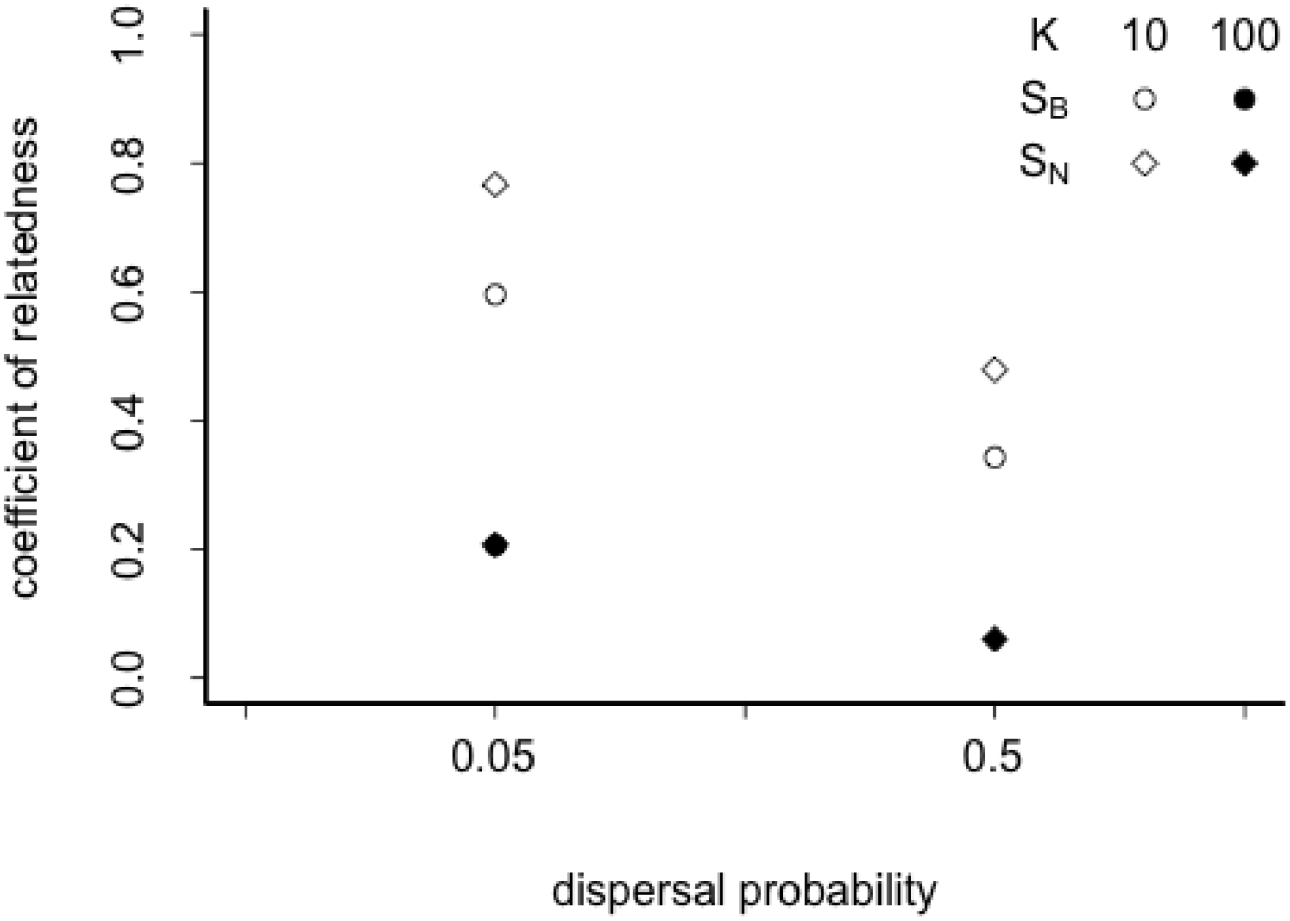
The effect of patch size (K), dispersal probability and dispersal strategy (S_B_, S_N_) on coefficient of relatedness after 20 generations. Different strategies S_B_ and S_N_ are represented by circles and diamond symbols, respectively. Small patch sizes (*K* = 10) are depicted with open symbols and big patch sizes (*K* = 100) with filled symbols.

The coefficients of relatedness (*F*) calculated for theadditionalnon-evolutionary simulations with fixed genetic traits of all individualsfurther confirm our speculations that breeding dispersal strategy is more advantageous avoiding kin competition. Thereby, we obtained significantly lower*F*after 20 generations values for breeding dispersal strategy when patches are small, while in big patches a difference is hardly noticeable(Fig 3). This corresponds with the stronger selection for S_B_we observe in metapopulations with small patches, while in big patches the two strategies become more similar. Coefficients for relatedness calculated after 5 generations yield higher values, however qualitatively are the same as those after 20 generations.

This effect is qualitatively similar, independent of whether we consider density-independent or density-dependent emigration (Fig 2). However, we observe the evolution of higher dispersal under density-independent compared to density-dependent scenario. This is not surprising because under density-independent scenario individuals disperse regardless of the patch density., thus risking to leave perfectly good habitat patch (patch of low density) while this is avoided under density-dependent emigration [42, 43]. Density dependence is thus more efficient in homogenizing fitness expectations between the patches. The discrepancy between DI and DD is especially large for low dispersal mortality and natal dispersal but it is considerably smaller under breeding dispersal.

#### Effect of carrying capacity and environmental variability

In the natal dispersal scenario we observe an increase in dispersal rates when reducing patch sizefrom*K* = 100 to *K* = 10(Fig 2). Smaller carrying capacity selects for increased dispersal because small *K* speeds the establishment of kin-structure and consequently intensifies kin competition [5, 8, 10, 55]. On the other hand, in the breeding dispersal scenario increasing mean carrying capacity from *K* = 10 to *K* = 100 leads to a noticeable increase in dispersal, as long as costs of dispersal are low (Fig 2). In the case of small *K* our simulations suggest that the reduction of sibling competition is the dominant effect for the evolution of dispersal—the introduction of considerable environmental variability (*σ* = 10 instead of *σ* = 0) thus results only in a rather weak increase in dispersal for the breeding dispersal strategy as compared to its effect on natal dispersal. It is important to realize that with small patch capacities the offspring of a single parent have a noticeable effect on population density and thus competition in that patch in general (not just on that between siblings) because the total number of individuals in the patch is so small; for this reason it remains a good strategy to distribute offspring rather evenly over two patches even if environmental variability is small. However, with large *K* the offspring of a single individual contribute only marginally to the intensity of competition in a local population and consequently we see a much stronger increase in breeding dispersal in this scenario due to the effect of environmental variability.

However, for high dispersal costs the effect is reversed and we observe the evolution of more dispersal for *K* = 10 compared to the scenario with *K* = 100, i.e. there is a noticeable interaction between the effects of patch size and dispersal mortality on the evolution of dispersal. Such an interaction effect does not occur for natal dispersal as we witness a decline in dispersal as *K* is increased over the whole range of values for dispersal cost. Overall, evolved dispersal rates are thus more similar for natal and breeding dispersal for scenarios with *K* = 100 compared to the scenarios with *K* = 10 (Fig 2a).

**Fig 4:**
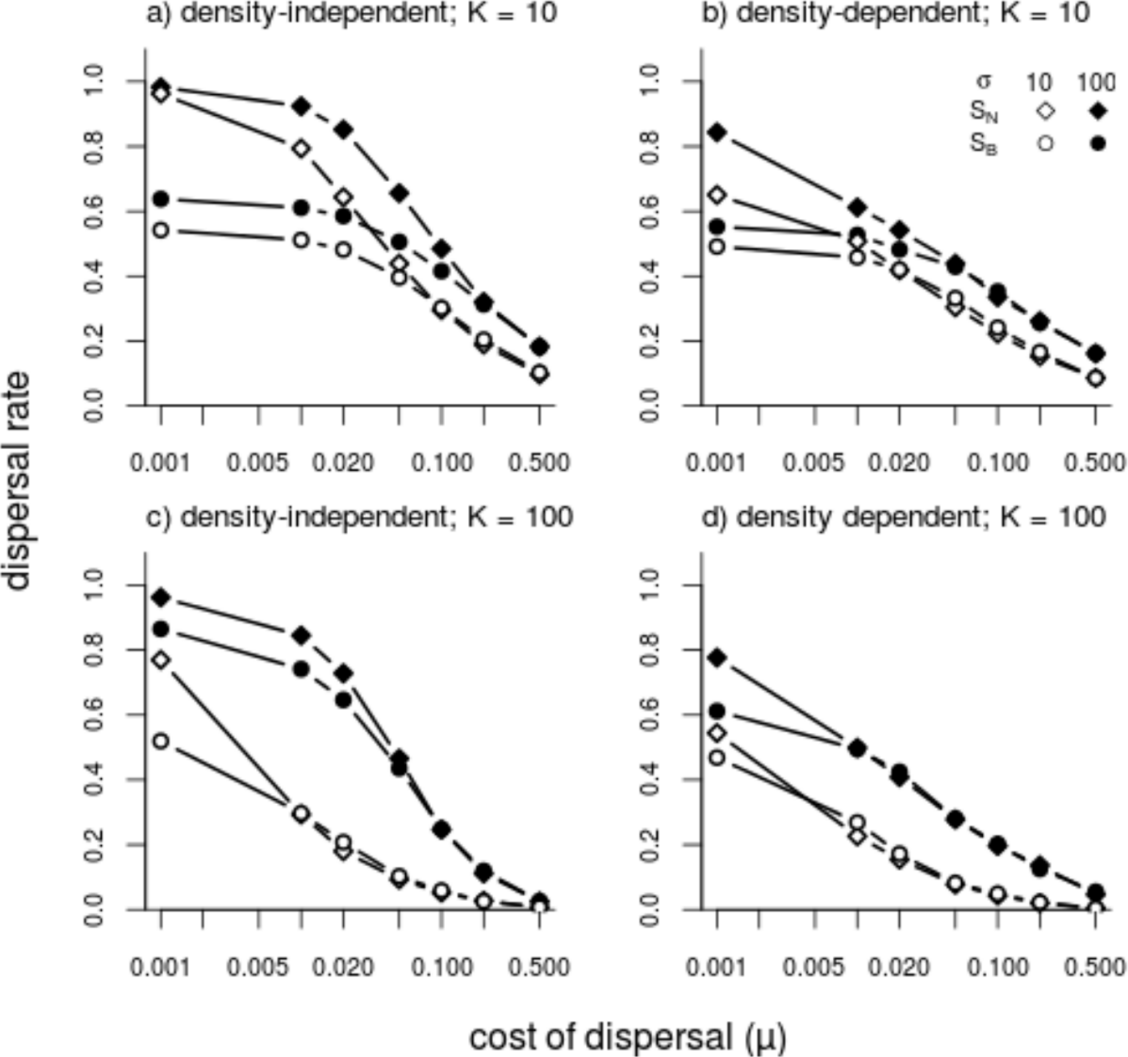
The effect of environmental variance (*σ*)), patch capacity (*K*) and dispersal costs (*μ*) on evolved dispersal rates. Density-independent (DI) dispersal (graphs a,c) and density-dependent (DD) dispersal (graphs b,d). Small carrying capacity (*K* = 10, graphs a,b) and big carrying capacity (*K* = 100, graphs c,d). Empty symbols (*σ* = 0) and filled symbols (*σ* = K). Diamonds and circles stand for S_N_ and S_B_ respectively.

Making populations demographically more stable by reducing environmental variability σ to zero has no qualitative effect on the results mentioned above (Fig 4). Especially for *K* = 10 we witness only a small to moderate reduction in evolving dispersal. For larger populations (*K* = 100) the decline in dispersal is more dramatic, especially in the intermediate range of values for dispersal costs. Here dispersal is mainly driven by environmental variability where as with K = 10 kin competition is a dominant factor [18].

### Evolutionary tournament

**Fig 5:**
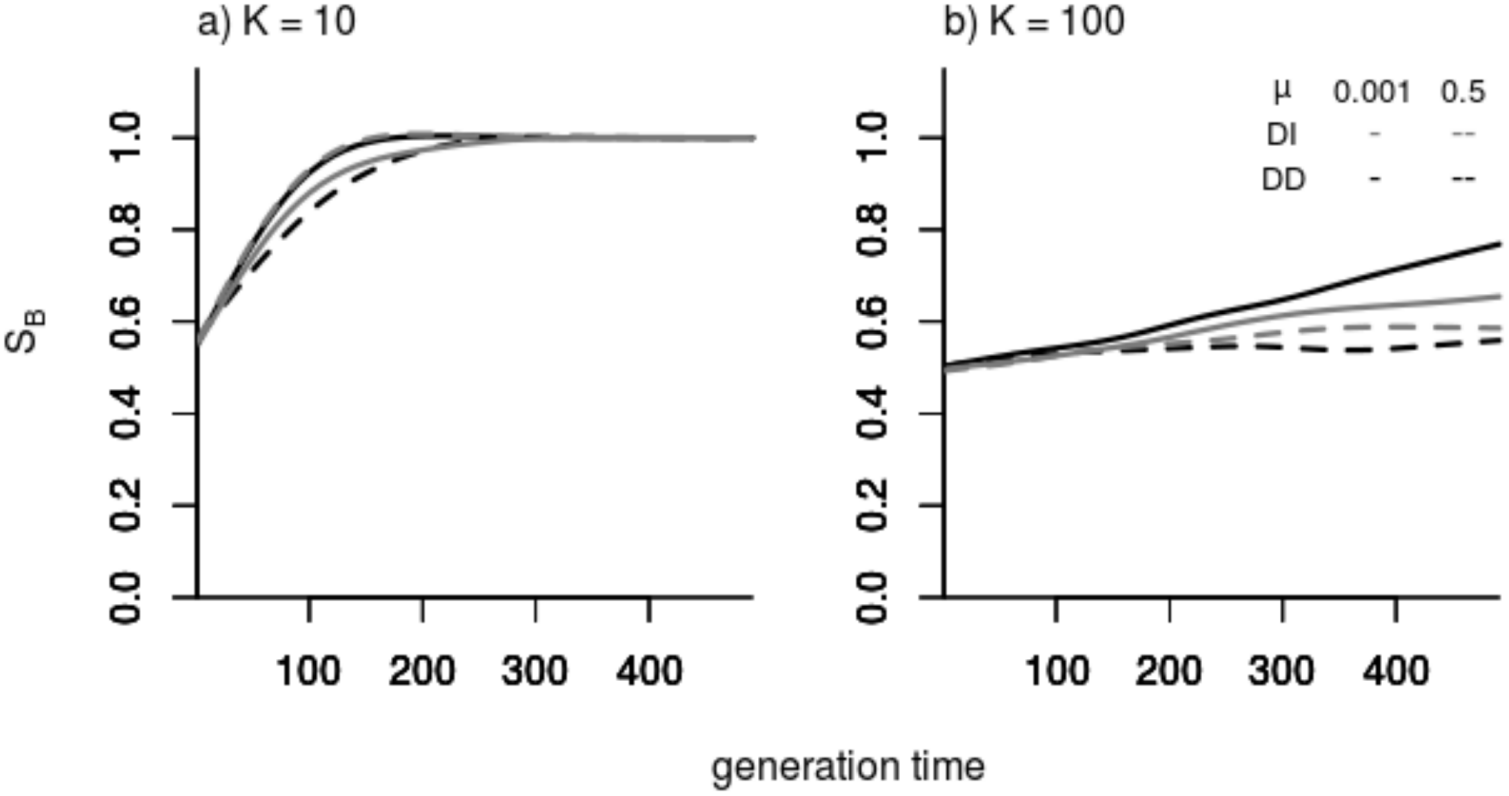
Exemplary change in the proportion (S_B_/(S_B_+S_N_)) of individuals with the breeding dispersal strategy over time during evolutionary tournaments. (a) Scenario with small patch size (*K* = 10) and (b) scenarios with large patch size (*K* = 100); environmental variability σ = *K* in all cases. Black and grey lines represent density-dependent (*DD*) and density-independent (*DI*) emigration scenarios, full and dashed lines represent mortalities *μ* = 0.001 and 0.5, respectively.

Comparing the resulting dispersal rates for the natal and breeding dispersal scenario, as such, does not allow deciding whether one strategy would prevail over the other in direct evolutionary competition. The evolutionary tournament where both strategies compete directly with each other allows doing that—and the results of the experiments are unambiguous: In all contests breeding dispersal out-competes natal dispersal. This is even true in a parameter range where evolved dispersal is similar for both strategies, i.e. at high dispersal costs (*μ* = 0.5). However, in a scenario with large patch size (*K*) and highenvironmental variability (σ)where strategies tended to evolve very similar dispersal rates (cf. Fig 2), replacement of the natal dispersal strategy progressed much slower than in scenarios where evolved emigration rates were dissimilar (Fig 5).

## Discussion

Our simulations confirm previous studies with respect to the effect of changes in dispersal costs [56] and environmental variability [19]. The effects of these variables can be understood by their effect on the costs and benefits of dispersing: The dispersal reducing effect of increased dispersal costs is obvious [9, 18, 50]. Environmental variability in turn enhances the formation of spatio-temporal variability in fitness expectations, which is known to promote dispersal [57]. The principal effects of these parameters on dispersal hold whatever dispersal scenario we implement: Neither a change from density-dependent to density-independent nor from natal to breedingdispersal undermines these general conclusions.

As confirmed in the natal dispersal scenarios, dispersal increases with decreasing patch carrying capacity [19]. Accordingly,higher emigration from smaller patches has indeed been observed for manybutterfly species in the field [58, 59, 60, 61, 62]. However, for breeding dispersal results are less clear in this respect: In the absence of environmental variability (*σ* = 0) the decline in dispersal with increasing *K* is rather weak. And we even observe an increase in dispersal with increasing patch capacity (*K*) for low dispersal costs in the scenarios with environmental variability. This is clearly contradicting the theoretical findings mentioned above that were always based on the assumption of natal dispersal, however. We will return to this interesting effect further below.

We should first note, that we generally see an apparent difference between natal and breeding dispersal only when dispersal costs become small: In all these scenarios, breeding dispersal evolves towards lower dispersal rates than natal dispersal and the fraction of offspring dispersed does typically not raise much beyond 0.5 (except if *K* = 100 and environment is variable). In contrast, under natal dispersal we see the evolution of emigration probabilities approaching one as dispersal costs approach zero; this is in good agreement with the predictions of Hamilton and May (1977).

To understand this discrepancy we have to consider the difference between the two strategies concerning their effect on kin and more specifically on direct sibling competition: Whatever the decision a 'natal disperser' takes—all of its offspring will always be born in a single patch. Under this strategy it is just impossible to reduce direct competition among siblings by dispersing. However by evolving a highly dispersive lineage, natal dispersal can reduce long-term 'trans-generational' kin competition, i.e. competition between grand-children and further descendants. In contrast, in the breeding dispersal strategy, dispersing parent can distribute offspring equally among two different patches and reduce kin competition among siblings already in the next generation.

Breeding dispersal out-competes natal dispersal under all conditions tested in our evolutionary tournaments, even where evolved dispersal rates are very similar and low. The previous argument concerning sibling competition is seemingly undermined at very low dispersal rates because in this case both strategies produce offspring more less in a same patch. Reducing direct sibling competition is, however, not the only benefit of the breeding over natal dispersal. A further, non-exclusive argument in favor of this strategy is that of 'risk spreading' [63]. In theory, distribution of offspring across different habitat patches with differing fitness expectations is thought to improve persistence in meta-populations. More precisely, by distributing offspring over several patches a parent may reduce the variance in the number of grandchildren produced as own offspring in different patches reproduce under different density conditions. Such a variance reducing effect should be beneficial as it increases the long-term geometric growth rate; and it is this rate that should ultimately be maximized by natural selection [46, 64]. Increasing the size of habitat patches (more precisely increasing the population size) shifts the balance from avoiding sibling competition in the next generation to the more long-term benefit of risk-spreading and promotes the evolution of more similar dispersal for the natal and breeding dispersal strategies. It is this shifting that is responsible for the increase in emigration rates in breeding dispersal above 0.5 whereas under natal dispersal it rather declines. Nonetheless, due to the benefits mentioned, breeding dispersal out-competes natal dispersal in all scenarios tested.

We as well as the other studies [42, 43] observe lower emigration rates under informed dispersal (Fig 2). The effect and benefits of informed, i.e. density-dependent emigration has been discussed before [27, 29, 43]. Fitness expectations are more efficiently (that means with fewer dispersal events) homogenized [26] across the landscape and overall lower net-dispersal evolves. This effect is especially valid in scenarios principally selecting for high dispersal, i.e. natal dispersal at low dispersal mortality. As a consequence we recognize that in the density-dependent scenarios dispersal becomes more similar between natal and breeding dispersal.

We should also note that the optimal residence time of *t*_*E*_ = 0.5 critically depends on the assumption that any individual is allowed to disperse at most once during its life-cycle. If we would allow for repeated dispersal during the reproductive season between several patches, leaving smaller fractions of offspring in each patch visited could clearly reduce sibling competition even further. We should consequently observe evolution of shorter patch residence times (smaller *t*_*E*_ values) in such a scenario.

Despite having clear theoretical benefits, mid-season breeding dispersal as we assume in this paper would not be an evolutionary option for organisms that care for their offspring, especially if that requires a stationary nest-site or territory. Yet for organisms like most insects or other annual organisms that typically do not show such behavior and that live in populations distributed in fragmented landscapes and unstable environments [65], breeding dispersal behavior could also be favored for additional reasons than those introduced here. Firstly, if an individual emerged in a certain patch, it might infer that it is a good quality patch. Thus, fitness-wise, the individual could benefit from staying some time and exploring the patch possibly leaving part of its offspring there. Secondly, it has been shown for some butterflies that older females tend to be more mobile than younger ones [66]. This is possibly due to the fact that older females already oviposited part of their egg-load and therefore become more agile in flight—increased mobility presumably increases the chance of successful dispersal. A study with spruce budworm moth species [67] shows that there is an oviposition threshold of around 50% in the natal patch, before females emigrate. Finally, informed emigration strategies where emigration decisions depend on population density or other attributes of patch quality demand that individuals acquire information about such attributes. Information acquisition, however, is itself a time-consuming process and it may be a rational decision to already deposit eggs while collecting information [27].

Empirical and theoretical work—especially that related to the investigation of insects or other annual organisms has paid little attention to the subtle difference between natal and breeding dispersal; the typical assumption in fact is that dispersal is natal. Our study shows that natural selection may generally favor the evolution of breeding dispersal in patchy environments and that evolving dispersal rates may quantitatively differ depending on which strategy an organism applies. It may be worth in future field studies of insects or other, similar organisms, to pay more attention to this difference and more carefully define at what moment in their life-time an individual dispersed.
